# Association of day of the week with mortality after elective right hemicolectomy for colon cancer: Case analysis from the National Clinical Database

**DOI:** 10.1002/ags3.12420

**Published:** 2021-01-15

**Authors:** Hiromichi Maeda, Hideki Endo, Nao Ichihara, Hiroaki Miyata, Hiroshi Hasegawa, Kinji Kamiya, Yoshihiro Kakeji, Kazuhiro Yoshida, Yasuyuki Seto, Hiroki Yamaue, Masakazu Yamamoto, Yuko Kitagawa, Sunao Uemura, Kazuhiro Hanazaki

**Affiliations:** ^1^ Department of Surgery Kochi Medical School Nankoku Japan; ^2^ Department of Healthcare Quality Assessment Graduate School of Medicine The University of Tokyo Bunkyo‐ku Japan; ^3^ Project Management Subcommittee The Japanese Society of Gastroenterological Surgery Tokyo Japan; ^4^ Database Committee The Japanese Society of Gastroenterological Surgery Tokyo Japan; ^5^ Department of Surgical Oncology Graduate School of Medicine Gifu University Gifu Japan; ^6^ Department of Gastrointestinal Surgery Graduate School of Medicine The University of Tokyo Bunkyo‐ku Japan; ^7^ Second Department of Surgery School of Medicine Wakayama Medical University Wakayama Japan; ^8^ Department of Surgery Institute of Gastroenterology Tokyo Women's Medical University Shinjuku‐ku Japan; ^9^ The Japanese Society of Gastroenterological Surgery Tokyo Japan

**Keywords:** colectomy, colonic neoplasms, elective surgical procedures, hospital mortality, treatment outcome

## Abstract

**Aim:**

We aimed to investigate whether later weekdays are related to worse short‐term outcomes after elective right hemicolectomy for colon cancer.

**Methods:**

We retrospectively analyzed adult patients who underwent elective right hemicolectomy for colon cancer between 2012 and 2017. Records lacking details about surgical mortality were excluded, and multiple imputation was performed for other missing data (variables). The primary endpoint was surgical mortality, defined as the sum of 30‐day mortality and in‐hospital deaths within 90 days postoperatively. Using 22 clinical variables, hierarchal logistic regression modeling with clustering of patients from the same institutes was performed.

**Results:**

Of the 112 658 patients undergoing elective right hemicolectomy for colon cancer, the 30‐day mortality and surgical mortality were 0.6% and 1.1%, respectively. Surgery on Friday was less frequent, accounting for 17.1% of all cases. The occurrence of severe postoperative complications, anastomotic leakage, or unadjusted odds ratio for surgical mortality did not show significant differences between weekdays. A hierarchal logistic regression model identified 19 independent factors for surgical mortality. Adjusted odds ratios for surgical mortality were 1.01 (95% confidence interval: 0.83‐1.22, *P* = .915), 0.86 (95% confidence interval: 0.71‐1.05, *P* = .144), 0.86 (95% confidence interval: 0.71‐1.05, *P* = .408), and 0.83 (95% confidence interval: 0.68‐1.03, *P* = .176) for Tuesday, Wednesday, Thursday, and Friday, respectively, showing no significant differences.

**Conclusion:**

This study did not identify an evident difference in surgical mortality between weekdays; a safe elective right hemicolectomy for colon cancer is being offered throughout the week in Japan.

## Graphical abstract

Weekday effect on mortality.

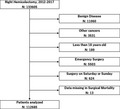

## 1 INTRODUCTION

In England, North America, and Northern Europe, the association between outcomes and the day of hospital admission or medical procedures has been investigated for decades.^1^, ^2^, ^3^, ^4^, ^5^ Elective surgery, a highly invasive medical procedure, drew extensive attention after a study based on administrative data of over 4.1 million cases revealed a steady increase in the odds ratio (OR) for postoperative mortality from Monday to Friday.^6^ In several societies, fewer medical resources are available on weekends. When surgeries are performed on Thursday or Friday, an unstable period early after the operation^7^, ^8^ coincides with the weekend, leading to worse prognosis due to ineffective response to adverse events.^3^ Fatigue is a known cause of performance impairment^9^ and may accumulate toward Friday, with the accuracy of surgery dropping at the peak of fatigue. Although the explanations sound real, rigid evidence of causative relationship is lacking. Furthermore, the limitation of risk adjustment due to the use of administrative data and integration of different operative procedures for various conditions remains a major issue of previous studies.^1^, ^2^, ^3^, ^4^, ^5^, ^6^, ^10^, ^11^

Colon and rectal cancers are a worldwide health problem,^12^ with right‐sided colon cancer accounting for 30%‐60% of all colon cancers.^13^, ^14^, ^15^ Between 2011 and 2017, the 30‐day postoperative mortality of elective/non‐elective right hemicolectomy was 1.1%‐1.3% while that of low anterior resection was 0.3%‐0.5% in Japan.^16^ The procedure with nodal dissection necessitates ligation of the right colic arteries and veins, and frequent variations are the cause of surgical difficulties and a risk of intraoperative hemorrhage.^17^ As ileo‐colic anastomosis (unlike anastomosis in pelvis) is located in the right upper abdomen, the intestinal content could widely scatter in the peritoneal cavity when anastomotic leakage occurs. As these severe complications jeopardize patients' lives and reduce the possibility of cure for disease,^18^, ^19^ offering safe surgery is one of the main goals of surgeons. To date, despite the numerous efforts to extract patient risk factors for short‐term outcomes of elective right hemicolectomy, a study investigating the effect of day of the week that surgery was performed on the outcomes is absent.

To elucidate the existence and the cause of disparities of short‐term outcomes after surgeries between different days of the week, “fine‐grained clinical data”^11^ are indispensable. Apart from administrative data, we decided to use data from the National Clinical Database (NCD) of Japan,^16^ which harbors relevant clinical data, including tumor, node, metastasis (TNM) classification, preoperative relevant variables, and postoperative complications. Data from the NCD represent more than 90% of the individual procedures performed in Japan,^20^ and their accuracy is assured.^21^ We considered that surgeons preferred to allocate surgeries with reduced risk to Fridays. Accordingly, our hypothesis was that the mortality before risk adjustment was similar throughout the week or slightly lower on Friday, and the mortality after risk adjustment is higher on Friday than on Monday. To our knowledge, the present study is the first large‐scale study investigating the effect of the day of the week on mortality focusing on elective right hemicolectomy for colon cancer instead of using a heterogenous group of patients.^22^, ^23^, ^24^, ^25^

## 2 METHODS

### 2.1 Patients and ethical approval

Patients who underwent right hemicolectomy between 1 January 2012 and 31 December 2017 were enrolled. Patients who underwent surgery due to non‐malignant disease or malignancies other than colon cancer were excluded. Next, patients who were aged <18 years, underwent emergency operations, underwent surgery on Saturday or Sunday, and those who lacked information concerning surgical mortality were excluded. Based on the database registration, emergency surgery was defined as a surgery performed within 24 hours after a decision was made because patients' lives or physical functions could be seriously damaged without operations. Surgeries performed on statutory holidays were not excluded because we considered that no practical difference would be observed by including this small fraction of patients. The protocol of this study was approved by the Japanese Society of Gastrointestinal Surgery committee, Japanese Society of Hepato‐Biliary‐Pancreatic Surgery committee, and ethical approval was obtained from the institutional review board of Kochi Medical School. Individual written informed consent was waived due to the retrospective design of this study.

### 2.2 Statistical methods

The primary endpoint of this study was surgical mortality after elective right hemicolectomy for colon cancer. Surgical mortality was defined as the sum of in‐hospital deaths within 90 days and 30‐day mortality. The 30‐day mortality was defined as any death occurring within 30 days after surgery. Other outcomes included operation time, estimated blood loss, severe postoperative complications, anastomotic leakage, and 30‐day mortality. Severe postoperative complication was defined as any postoperative surgical and medical complication with a Clavien‐Dindo classification of III or more.

For risk adjustment, we used the year of surgery, age at surgery, sex, body mass index, activities of daily living, American Society of Anesthesiologists Performance Status classification,^26^ Union for International Cancer Control‐TNM classification (7th edition),^27^ surgical approach (open or laparoscopy), white blood cell count, platelet count, prothrombin time‐international normalized ratio, activated partial thromboplastin time (APTT), blood urea nitrogen, creatinine, absence/presence of chronic obstructive pulmonary disease, dyspnea, recent history of weight loss (more than 10% within 6 months), and bleeding disorder (including anticoagulant use).

To compare baseline characteristics and short‐term outcomes, the chi‐squared test was used for categorical variables, whereas the Kruskal‐Wallis test was performed for variables with continuous values. For missing data of variables, multiple imputation was performed.^28^, ^29^ The ORs for Tuesday to Friday were calculated by setting Monday as the reference. A hierarchal logistic regression model was used to calculate the ORs for surgical mortality, with consideration of the correlation within the cluster composed of patients from the same institutes.^30^ *P* values <.05 were considered statistically significant. All statistical analyses were performed using R version 3.6.1 (2019; R Foundation for Statistical Computing).

## 3 RESULTS

### 3.1 Patient characteristics and surgery

**FIGURE 1:**
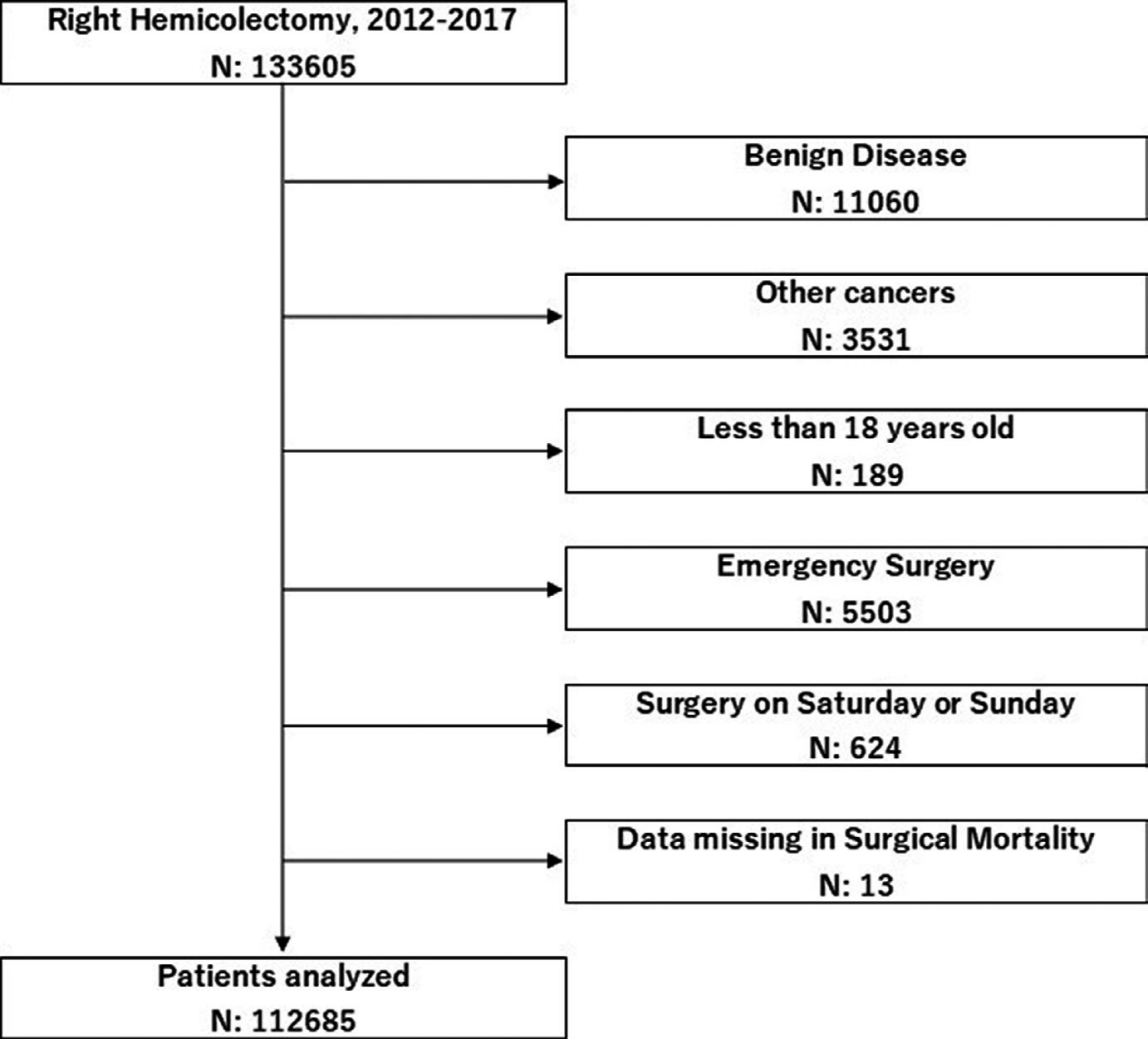
Inclusion and exclusion criteria for the patient

In total, data of 112 658 patients were chosen from a potential sample of 133 605 patients (Figure 1). Of them, 50.5% and 49.5% were women and men, respectively. The number of registered patients gradually increased from 2012 to 2015. Patients aged over 80 years accounted for approximately 30% of patients. Furthermore, 75.9% of patients had T3/T4‐stage tumors, 44.7% had lymph node metastasis, and 12.5% had synchronous metastatic lesions (Table S1).

Surgeries were most frequently performed on Monday, followed by Wednesday. Surgeries performed on Friday accounted for 17.1% of all cases, which was 30.4% fewer than surgeries performed on Monday. After stratifying patients according to the day of the week, year of surgery, age, Brinkman index, T‐factor, level of APTT, and serum creatinine levels showed statistically significant differences (Table S2).

### 3.2 Surgical outcomes

Overall 30‐day mortality was 0.6%, ranging from 0.5% to 0.7% throughout the weekdays without statistically significant differences (Table 1). Likewise, surgical mortality was 1.1%, ranging from 1.0% to 1.2%, and was not significantly different. Severe postoperative complications, anastomotic leakage, and operation time showed no significant differences between the days of the week. The estimated blood loss was 67 mL on Monday and 60 mL on Friday, showing a significant difference (*P* < .05).

**TABLE 1:** Short‐term operative outcomes

	Monday (n = 25 425)	Tuesday (n = 22 096)	Wednesday (n = 24 001)	Thursday (n = 21 906)	Friday (n = 19 257)	*P* value
Operation time, min						
Median [IQR]	197.0 [151.0, 254.0]	198.0 [151.0, 255.0]	199.0 [152.0, 256.0]	197.0 [152.0, 254.0]	199.0 [152.0, 254.0]	.06
Estimated blood loss, mL						
Median [IQR]	67.00 [20.0, 187.5]	62.0 [20.0, 180.0]	65.00 [20.0, 183.0]	60.0 [20.0, 178.0]	60.0 [20.0, 180.0]	<.001
Severe complication						
Yes, n (%)	1216 (4.8)	1070 (4.8)	1107 (4.6)	954 (4.4)	888 (4.6)	.12
No, n (%)	24 207 (95.2)	21 022 (95.2)	22 888 (95.4)	20 950 (95.6)	18 365 (95.4)	
Anastomotic leakage						
Yes, n (%)	402 (1.6)	339 (1.5)	380 (1.6)	347 (1.6)	301 (1.6)	.99
No, n (%)	25 018 (98.4)	21 754 (98.5)	23 617 (98.4)	21 555 (98.4)	18 952 (98.4)	
30‐Day mortality						
Yes, n (%)	156 (0.6)	147 (0.7)	121 (0.5)	120 (0.5)	95 (0.5)	.08
No, n (%)	25 269 (99.4)	21 949 (99.3)	23 879 (99.5)	21 786 (99.5)	19 162 (99.5)	
Surgical mortality						
Yes, n (%)	281 (1.1)	258 (1.2)	235 (1.0)	235 (1.1)	189 (1.0)	.23
No, n (%)	25 144 (98.9)	25 144 (98.9)	23 766 (99.0)	21 671 (98.9)	19 068 (99.0)	

Note

*P* values concern the overall comparison, not the comparison between individual days of the week.

Abbreviation: IQR, interquartile range.

### 3.3 Unadjusted ORs

The unadjusted ORs for surgical mortality were 1.06 (95% confidence interval [CI]: 0.89‐1.25) for Tuesday, 0.89 (95% CI: 0.74‐1.05) for Wednesday, 0.97 (95% CI: 0.82‐1.16) for Thursday, and 0.89 (95% CI: 0.74‐1.07) for Friday (Table 2). No statistically significant difference was observed when they were individually compared to Monday.

**TABLE 2:** Unadjusted and adjusted odds ratios for surgical mortality

Operative day	Odds ratio [95% CI]	*P* value
Unadjusted		
Monday	1	
Tuesday	1.06 [0.89‐1.25]	.52
Wednesday	0.89 [0.74‐1.05]	.17
Thursday	0.97 [0.82‐1.16]	.73
Friday	0.89 [0.74‐1.07]	.20
Adjusted		
Monday	1	
Tuesday	1.01 [0.83‐1.22]	.92
Wednesday	0.86 [0.71‐1.05]	.14
Thursday	0.86 [0.71‐1.05]	.41
Friday	0.83 [0.68‐1.03]	.18

Note

*P* value was determined for comparisons using Monday as the reference.

Abbreviation: CI, confidence interval.

### 3.4 Adjusted ORs

Hierarchal logistic regression modeling revealed 19 factors associated with surgical mortality after elective right hemicolectomy (Table S3). Among these factors, four clinical variables demonstrated differences of the distributions among the days of week. Patients aged more than 75 years and tumors with T3/4 were more abundant on Monday compared to the rest of the weekdays (Table S2). The adjusted ORs for surgical mortality were 1.01 (95% CI: 0.83‐1.22) for Tuesday, 0.86 (95% CI: 0.71‐1.05) for Wednesday, 0.86 (95% CI: 0.71‐1.05) for Thursday, and 0.83 (95% CI: 0.68‐1.03) for Friday, showing no significant difference (Table 2).

## 4 DISCUSSION

In this retrospective analysis using the clinical data of 112 685 patients, we found no evidence suggesting that patients undergoing elective right hemicolectomy for colon cancer on Friday were at higher risk of mortality. Furthermore, the unadjusted OR for mortality, frequency of severe postoperative complications, or operation time did not reveal any significant difference when stratified according to the days of the week.

Our study has several characteristic features pertaining to the results. First, the 30‐day mortality and surgical mortality were as low as 0.6% and 1.1%, respectively. In another study using the NCD, the 30‐day mortality after low anterior resection of the rectum was 0.3%‐0.5%,^19^ which also included emergency cases. In contrast, the crude 30‐day mortality for elective colorectal (colon and rectum) surgery in previous studies was approximately 2.2%‐3.5%.^6^, ^22^, ^23^, ^24^ The low frequency of events (death) could have simply impeded the detection of a small difference in mortality between days of the week despite the large sample size in the present study.

Second, the unadjusted and adjusted OR for surgical mortality on Friday was lower than that found on early days of the week (Monday/Tuesday), although a statistical significance was not apparent. In contrast, the higher unadjusted mortality on Friday/weekend has been demonstrated in previous studies.^6^, ^23^, ^31^ Their result suggests that the disparity in medical care quality across different days of the week was quite considerable, especially when patients undergoing surgery on later of the weekday had fewer comorbidities.^6^ However, the concern is the high mortality (57.3/1000; 5.7%) among patients undergoing elective colorectal surgeries on the weekend,^6^ which was almost equivalent to that for emergency right hemicolectomy performed in Japan.^32^ This implies that emergency surgery or semi‐emergency surgery should be better defined, and the contamination of emergency surgery cases with semi‐emergencies has to be monitored.

Third, our study appropriately incorporated the acuity of the disease (TNM classification) and general variables obtained from the clinical database into risk adjustment. Conversely, the unreliability of risk adjustment in previous studies with administrative data has been raised,^1^, ^10^ and the appropriateness of the predictive ability between clinical and administrative data remains controversial,^33^ together with the inconsistency of the commonly used Charlson comorbidity index and other scores.^34^ Moreover, only patients undergoing elective right‐hemicolectomy for colon cancer were included in the present study. Operation methods, morbidity, and mortalities of surgeries for colon and rectum are distinct.^16^ Furthermore, the association between quality of care and the results differs among diseases,^11^ and we consider that these conditions (including right‐ and left‐sided colon cancers) should be examined separately. Because of the consistent approach to the data analysis in the present study, we consider our results being robust and conclusive.

A possible explanation for our findings has already been proposed by Galyfos et al in their meta‐analysis for elective vascular surgery.^35^ The authors revealed that studies from the United States and the United Kingdom, where surgical treatment is performed in centralized institutes, have demonstrated the effect of day of the week on mortality. Assumingly, similar institutional systems or practice patterns accentuate the difference between days of the week when cases are accumulated. In contrast, they claimed that data from Canada, where decentralized health system is characteristic, indicated no evidence of relationship between days of the week and short‐term outcomes. Thus, the diverse practice pattern disperses the effect of the days of the week when cases are accumulated, even if disparities in the quality of medical care between days of the week may exist. We considered that since the health care system in Japan is decentralized, the theory proposed by Galyfos et al^35^ could be applied. Centralization of surgery is a trend in Japan^36^ and monitoring the effect of the days of the week would be important. Meanwhile, the theory may not be supported by a recent study focusing on the cardiovascular surgery performed in 10 high‐volume centers in the United Kingdom, which demonstrated no evidence of weekend effect on mortality.^37^

A more compelling explanation for the results is simply that the same quality level of surgeries and care was provided throughout the week. The high percentage of participation by board‐certificated surgeons might represent the rich infrastructure and resources of the medical system in Japan. Approximately 75% of surgeries targeting the small intestine and colon are performed by Japanese Society of Gastrointestinal Surgery board‐certified training institutes.^16^ Up to 70% of surgical procedures involve the participation of board‐certificated surgeons (as primary surgeons or assistant surgeons), and the rate of surgeries performed by these surgeons is 25%‐30%,^16^ which is considerably higher than the rate observed in the United States.^38^ The adequacy of the board certification system in the field of the esophagus^39^ may support our reflection. Furthermore, other factors including nursing skills, staff allocation, role of the anesthesiologist, and availability of surgical intensive care units should be addressed in the future because postoperative care is complex. Transactional analysis across different medical systems would clarify the significant influential factors for outcomes related to the day of the week.

The present study did not reveal any differences in terms of frequencies of severe morbidity, anastomotic leakage, or operation time between the days of the week. The estimated blood loss was slightly higher on Monday. The clinical implication of small differences with large volume data has to be contemplated individually, although we found no clinical significance/meaning for this finding. These secondary outcomes were analyzed from the standpoint of the speculation that fatigue or differences in staff allocation on Friday could negatively affect the quality of the operation itself. In retrospect, the failure‐to‐rescue rate would represent an additional analysis to evaluate the effectiveness of responses to adverse events.^40^ However, we refrained from any further analysis because it was not included in the initial study protocol and it was unlikely to identify differences in mortality and morbidity shown in the present study.

## 5 LIMITATIONS

This study had limitations. The results and interpretations should be limited to elective right hemicolectomy for colon cancer within the modern healthcare system in Japan. Namely, the extrapolation of our result to other surgical procedures should be avoided, even to the low anterior resection for rectal cancer. We acknowledge that the weekend (or weekday) effect may exist for certain procedures in other medical systems,^1^, ^41^ although it is not a universal phenomenon.^25^ In addition, we do not have the data concerning the cause of death, which may help us to understand the reasons for the lack of the relationship between days of week and surgical mortalities. For instance, if the anastomotic leakage is the most frequent cause of death in the present study, a conceivable claim is that its delayed occurrence after colorectal surgery (usually a few days after operation) may have obscured the effect of the days of the week. Finally, our findings do not necessarily mean that all procedures related to elective right hemicolectomy for colon cancer were performed under uniform conditions throughout the weekdays. As proposed,^41^ other quality indicators such as long‐term prognosis, number of dissected lymph nodes, and patient‐oriented evaluations could better elucidate the disparity in medical care quality between the days of the week.

Although a more prudent analytic approach can assess the effect of the days of the week on postoperative outcomes, we consider that appropriate risk adjustment using clinical data is inevitable. As the impact of treatment on prognosis differs among diseases, an individual analysis of each specific procedure should be performed for each area/country sharing the same medical system. The present study, designed to reflect these considerations, showed that there was no evidence of a relationship between days of the week and mortality after elective right hemicolectomy for colon cancer. Considering a surgical mortality of 1.1% (30‐day mortality plus 90‐day in‐hospital deaths), this procedure is being safely performed throughout the weekdays in Japan.

## DISCLOSURE

Funding: Part of the research expense was supported by the Japanese Society of Hepato‐Biliary‐Pancreatic Surgery.

Conflicts of Interest: HE, NI, and HM are affiliated to the Department of Healthcare Quality Assessment at the University of Tokyo. The department is a social collaboration department supported by grants from the National Clinical Database, Johnson & Johnson KK, and Nipro Corporation. YK was supported by grants or donation from Taiho Pharmaceutical Co., Ltd and Chugai Pharmaceutical Co., Ltd., received lecture fees from Asahi Kasei Co. Ltd, Taiho Pharmaceutical Co., Ltd and Chugai Pharmaceutical Co. Ltd., and holds an endowed chair by Taiho Pharmaceutical Co. Ltd and Chugai Pharmaceutical Co. Ltd.

## ETHICAL APPROVAL

The protocol of this study was approved by the Japanese Society of Gastrointestinal Surgery committee, Japanese Society of Hepato‐Biliary‐Pancreatic Surgery committee, and the institutional review board of Kochi Medical School. Individual written informed consent was waived due to the design of this retrospective study.

## Supporting information

Table S1‐S3
