# Supplementary material for: Association of day of the week with mortality after elective right hemicolectomy for colon cancer: Case analysis from the National Clinical Database
Source: Ann Gastroenterol Surg. 2021 Jan 15;5(3):331–7. doi: 10.1002/ags3.12420 (PMC8164462; doi:10.1002/ags3.12420)
Supplement: Supplementary file 1 — Table S1‐S3 [file AGS3-5-331-s001.docx]

**Supporting Information**

| **Table 1. Clinical Characteristics of the Patients** | | | |
| --- | --- | --- | --- |
| **Variables** |  | **Overall (%)** | **Missing (%)** |
| All cases |  | 112685 |  |
| Day of the week | Monday | 25425 (22.6) | 0 |
|  | Tuesday | 22096 (19.6) |  |
|  | Wednesday | 24001 (21.3) |  |
|  | Thursday | 21906 (19.4) |  |
|  | Friday | 19257 (17.1) |  |
| Year | 2012 | 17708 (15.7) | 0 |
|  | 2013 | 18287 (16.2) |  |
|  | 2014 | 18863 (16.7) |  |
|  | 2015 | 19336 (17.2) |  |
|  | 2016 | 19380 (17.2) |  |
|  | 2017 | 19111 (17.0) |  |
| Age, years | 18–59 | 10961 (9.7) | 0 |
|  | 60–64 | 9890 (8.8) |  |
|  | 65–69 | 16350 (14.5) |  |
|  | 70–74 | 20235 (18.0) |  |
|  | 75–79 | 22035 (19.6) |  |
|  | 80– | 33214 (29.5) |  |
| Sex | Female | 56877 (50.5) | 0 |
|  | Male | 55807 (49.5) |  |
| BMI, kg/m^2^ | Normal (18.5 ≤ BMI < 25) | 73389 (65.2) | 0 |
|  | Underweight (BMI <18.5) | 16804 (14.9) |  |
|  | Overweight (BMI ≥25) | 22438 (19.9) |  |
| Activities of daily living | Independent | 104162 (92.4) | 0 |
|  | Dependent | 8512 (7.6) |  |
| COPD | No | 109149 (96.9) | 0 |
|  | Yes | 3521 (3.1) |  |
| Brinkman index | 0 | 81251 (72.1) | 0.1 |
|  | <400 | 9843 (8.7) |  |
|  | ≥400 | 21524 (19.1) |  |
| Dyspnea | No | 110458 (98.0) | 0 |
|  | Yes | 2213 (2.0) |  |
| Angina | No | 111171 (98.7) | 0 |
|  | Yes | 1495 (1.3) |  |
| Loss of body weight | No | 107704 (95.6) | 0 |
|  | Yes | 4967 (4.4) |  |
| Coagulation disorder | No | 108042 (95.9) | 0 |
|  | Yes | 4629 (4.1) |  |
| ASA_PS | ASA 1–2 | 96929 (86.0) | 0 |
|  | ASA 3–5 | 15749 (14.0) |  |
| T category | T0, Tis, 1, 2 | 26869 (23.9) | 0.2 |
|  | T3, 4a, 4b | 85429 (75.9) |  |
|  | TX | 201 (0.2) |  |
| N category | N0 | 61392 (54.6) | 0.2 |
|  | N1a, 1b, 1c, 2a, 2b | 50257 (44.7) |  |
|  | NX | 850 (0.8) |  |
| M category | M0 | 98498 (87.5) | 0.1 |
|  | M1a, M1b | 14044 (12.5) |  |
| White blood cell count, /μL | Normal (3500–9000) | 98569 (87.8) | 0.4 |
|  | Low (<3500) | 3602 (3.2) |  |
|  | High (>9000) | 10100 (9.0) |  |
| Platelet count, ×104/μL | Normal (15–35） | 91973 (81.9) | 0.4 |
|  | Low (<15) | 4988 (4.4) |  |
|  | High (>35） | 15272 (13.6) |  |
| PT-INR | Normal (0.9–1.1） | 94081 (87.2) | 4.3 |
|  | Low (<0.9) | 1901 (1.8) |  |
|  | High (>1.1) | 11876 (11.0) |  |
| APTT, sec | Normal (30–40) | 75862 (70.7) | 4.8 |
|  | Low (<30) | 27264 (25.4) |  |
|  | High (>40) | 4158 (3.9) |  |
| BUN, mg/dL | Normal (8–20) | 94375 (84.4) | 0.8 |
|  | Low (<8) | 4760 (4.3) |  |
|  | High (>20) | 12672 (11.3) |  |
| Creatinine, mg/dL | Normal (≤2) | 109779 (98.4) | 1 |
|  | High (>2) | 1755 (1.6) |  |
| Surgical approach | Open | 56802 (50.4) | 0 |
|  | Laparoscopic | 55883 (49.6) |  |
| Operation time, min (median [IQR]) |  | 198.00 [152.00, 255.00] | 0 |
| Estimated blood loss, mL (median [IQR]) |  | 64.00 [20.00, 180.00] | 0.1 |
| 30-Day mortality | No | 112045 (99.4) | 0 |
|  | Yes | 639 (0.6) |  |
| Surgical mortality | No | 111487 (98.9) | 0 |
|  | Yes | 1198 (1.1) |  |
| Morbidity with CD classification of III or more | No | 107432 (95.4) | 0 |
|  | Yes | 5235 (4.6) |  |
| Anastomotic leakage | No | 110896 (98.4) | 0 |
|  | Yes | 1769 (1.6) |  |

BMI, body mass index; COPD, chronic obstructive pulmonary disease; ASA_PS, American Society of Anesthesiologists Performance Status; PT-INR, prothrombin time-international normalized ratio; APTT, activated partial thromboplastin time; BUN, blood urea nitrogen; IQR, interquartile range; CD, Clavien-Dindo

| **Table 2. Distribution of the Patients by Day of the Week** | | | | | | | | | | | |
| --- | --- | --- | --- | --- | --- | --- | --- | --- | --- | --- | --- |
| **Variables** | **Level** | **Monday (%)** | **Tuesday (%)** | | **Wednesday (%)** | | **Thursday (%)** | | **Friday**  **(%)** | | ***P* value** |
| All cases |  | 25425 | 22096 | | 24001 | | 21906 | | 19257 | |  |
| Year | 2012 | 3946 (15.5) | 3426 (15.5) | | 3727 (15.5) | | 3484 (15.9) | | 3125 (16.2) | | <0.001 |
|  | 2013 | 3721 (14.6) | 3761 (17.0) | | 3826 (15.9) | | 3696 (16.9) | | 3283 (17.0) | |  |
|  | 2014 | 4305 (16.9) | 3389 (15.3) | | 4219 (17.6) | | 3709 (16.9) | | 3241 (16.8) | |  |
|  | 2015 | 4522 (17.8) | 3670 (16.6) | | 3873 (16.1) | | 3854 (17.6) | | 3417 (17.7) | |  |
|  | 2016 | 4563 (17.9) | 3979 (18.0) | | 4144 (17.3) | | 3508 (16.0) | | 3186 (16.5) | |  |
|  | 2017 | 4368 (17.2) | 3871 (17.5) | | 4212 (17.5) | | 3655 (16.7) | | 3005 (15.6) | |  |
| Age, years | 18–59 | 2421 (9.5) | 2113 (9.6) | | 2278 (9.5) | | 2211 (10.1) | | 1938 (10.1) | | 0.029 |
|  | 60–64 | 2190 (8.6) | 1953 (8.8) | | 2102 (8.8) | | 1882 (8.6) | | 1763 (9.2) | |  |
|  | 65–69 | 3622 (14.2) | 3190 (14.4) | | 3566 (14.9) | | 3104 (14.2) | | 2868 (14.9) | |  |
|  | 70–74 | 4488 (17.7) | 4056 (18.4) | | 4311 (18.0) | | 3955 (18.1) | | 3425 (17.8) | |  |
|  | 75–79 | 5078 (20.0) | 4276 (19.4) | | 4645 (19.4) | | 4314 (19.7) | | 3722 (19.3) | |  |
|  | 80– | 7626 (30.0) | 6508 (29.5) | | 7099 (29.6) | | 6440 (29.4) | | 5541 (28.8) | |  |
| Sex | Female | 12855 (50.6) | 11167 (50.5) | | 12121 (50.5) | | 11070 (50.5) | | 9664 (50.2) | | 0.938 |
|  | Male | 12569 (49.4) | 10929 (49.5) | | 11880 (49.5) | | 10836 (49.5) | | 9593 (49.8) | |  |
| BMI, kg/m^2^ | Normal (18.5 ≤ BMI < 25) | 16510 (65.0) | 14388 (65.2) | | 15695 (65.4) | | 14269 (65.2) | | 12527 (65.1) | | 0.814 |
|  | Underweight (BMI <18.5) | 3862 (15.2) | 3315 (15.0) | | 3510 (14.6) | | 3225 (14.7) | | 2892 (15.0) | |  |
|  | Overweight (BMI ≥25) | 5042 (19.8) | 4380 (19.8) | | 4784 (19.9) | | 4404 (20.1) | | 3828 (19.9) | |  |
| Activities of daily living | Independent | 23515 (92.5) | 20374 (92.2) | | 22228 (92.6) | | 20180 (92.1) | | 17865 (92.8) | | 0.052 |
|  | Dependent | 1908 (7.5) | 1720 (7.8) | | 1768 (7.4) | | 1726 (7.9) | | 1390 (7.2) | |  |
| COPD | No | 24661 (97.0) | 21387 (96.8) | | 23238 (96.8) | | 21214 (96.8) | | 18649 (96.9) | | 0.761 |
|  | Yes | 762 (3.0) | 705 (3.2) | | 758 (3.2) | | 691 (3.2) | | 605 (3.1) | |  |
| Brinkman index | 0 | 18352 (72.2) | 15915 (72.1) | | 17473 (72.8) | | 15827 (72.3) | | 13684 (71.1) | | 0.002 |
|  | <400 | 2139 (8.4) | 1920 (8.7) | | 2033 (8.5) | | 1975 (9.0) | | 1776 (9.2) | |  |
|  | ≥400 | 4914 (19.3) | 4246 (19.2) | | 4487 (18.7) | | 4094 (18.7) | | 3783 (19.7) | |  |
| Dyspnea | No | 24940 (98.1) | 21617 (97.9) | | 23562 (98.2) | | 21455 (97.9) | | 18884 (98.1) | | 0.079 |
|  | Yes | 484 (1.9) | 474 (2.1) | | 435 (1.8) | | 451 (2.1) | | 369 (1.9) | |  |
| Angina | No | 25062 (98.6) | 21822 (98.8) | | 23680 (98.7) | | 21616 (98.7) | | 18991 (98.6) | | 0.435 |
|  | Yes | 359 (1.4) | 269 (1.2) | | 317 (1.3) | | 286 (1.3) | | 264 (1.4) | |  |
| Loss of body weight | No | 24324 (95.7) | 21118 (95.6) | | 22915 (95.5) | | 20953 (95.7) | | 18394 (95.5) | | 0.836 |
|  | Yes | 1098 (4.3) | 976 (4.4) | | 1082 (4.5) | | 951 (4.3) | | 860 (4.5) | |  |
| Coagulation disorder | No | 24363 (95.8) | 21219 (96.0) | | 22995 (95.8) | | 21030 (96.0) | | 18435 (95.7) | | 0.481 |
|  | Yes | 1058 (4.2) | 875 (4.0) | | 1001 (4.2) | | 875 (4.0) | | 820 (4.3) | |  |
| ASA_PS | ASA 1–2 | 21855 (86.0) | 18963 (85.8) | | 20752 (86.5) | | 18788 (85.8) | | 16571 (86.1) | | 0.205 |
|  | ASA 3–5 | 3568 (14.0) | 3132 (14.2) | | 3247 (13.5) | | 3116 (14.2) | | 2686 (13.9) | |  |
| T category | T0, Tis, 1, 2 | 5794 (22.8) | 5278 (23.9) | | 5875 (24.5) | | 5329 (24.4) | | 4593 (23.9) | | 0.001 |
|  | T3, 4a, 4b | 19546 (77.0) | 16747 (75.9) | | 18027 (75.3) | | 16493 (75.4) | | 14616 (76.0) | |  |
|  | TX | 41 (0.2) | 38 (0.2) | | 50 (0.2) | | 41 (0.2) | | 31 (0.2) | |  |
| N category | N0 | 13796 (54.4) | 12086 (54.8) | | 13181 (55.0) | | 11898 (54.4) | | 10431 (54.2) | | 0.452 |
|  | N1a, 1b, 1c, 2a, 2b | 11400 (44.9) | 9814 (44.5) | | 10576 (44.2) | | 9812 (44.9) | | 8655 (45.0) | |  |
|  | NX | 187 (0.7) | 160 (0.7) | | 197 (0.8) | | 152 (0.7) | | 154 (0.8) | |  |
| M category | M0 | 22192 (87.4) | 19286 (87.4) | | 21031 (87.8) | | 19169 (87.6) | | 16820 (87.4) | | 0.617 |
|  | M1a, M1b | 3200 (12.6) | 2784 (12.6) | | 2932 (12.2) | | 2701 (12.4) | | 2427 (12.6) | |  |
| White blood cell count, /μL | Normal (3500–9000) | 22230 (87.7) | 19322 (87.8) | | 21097 (88.2) | | 19115 (87.6) | | 16805 (87.6) | | 0.48 |
|  | Low (<3500) | 799 (3.2) | 723 (3.3) | | 768 (3.2) | | 699 (3.2) | | 613 (3.2) | |  |
|  | High (>9000) | 2310 (9.1) | 1968 (8.9) | | 2057 (8.6) | | 2008 (9.2) | | 1757 (9.2) | |  |
| Platelet count, ×104/μL | Normal (15–35） | 20702 (81.7) | 17982 (81.7) | | 19661 (82.2) | | 17880 (82.0) | | 15748 (82.1) | | 0.417 |
|  | Low (<15) | 1123 (4.4) | 1015 (4.6) | | 1067 (4.5) | | 983 (4.5) | | 800 (4.2) | |  |
|  | High (>35） | 3505 (13.8) | 3011 (13.7) | | 3185 (13.3) | | 2947 (13.5) | | 2624 (13.7) | |  |
| PT-INR | Normal (0.9–1.1） | 21216 (87.2) | 18432 (87.2) | | 20048 (87.4) | | 18303 (87.3) | | 16082 (87.1) | | 0.738 |
|  | Low (<0.9) | 446 (1.8) | 345 (1.6) | | 410 (1.8) | | 367 (1.7) | | 333 (1.8) | |  |
|  | High (>1.1) | 2663 (10.9) | 2372 (11.2) | | 2481 (10.8) | | 2302 (11.0) | | 2058 (11.1) | |  |
| APTT, sec | Normal (30–40) | 17166 (70.9) | 14821 (70.6) | | 16379 (71.7) | | 14652 (70.3) | | 12844 (69.8) | | <0.001 |
|  | Low (<30) | 6085 (25.1) | 5330 (25.4) | | 5585 (24.5) | | 5370 (25.8) | | 4894 (26.6) | |  |
|  | High (>40) | 960 (4.0) | 845 (4.0) | | 871 (3.8) | | 815 (3.9) | | 667 (3.6) | |  |
| BUN, mg/dL | Normal (8–20) | 21417 (84.9) | 18457 (84.2) | | 20062 (84.2) | | 18322 (84.4) | | 16117 (84.3) | | 0.505 |
|  | Low (<8) | 1067 (4.2) | 936 (4.3) | | 1028 (4.3) | | 906 (4.2) | | 823 (4.3) | |  |
|  | High (>20) | 2750 (10.9) | 2524 (11.5) | | 2735 (11.5) | | 2491 (11.5) | | 2172 (11.4) | |  |
| Creatinine, mg/dL | Normal (≤2) | 24855 (98.7) | 21488 (98.3) | | 23386 (98.3) | | 21315 (98.3) | | 18735 (98.4) | | <0.001 |
|  | High (>2) | 317 (1.3) | 365 (1.7) | | 399 (1.7) | | 360 (1.7) | | 314 (1.6) | |  |
| Surgical approach | Open | 12950 (50.9) | 11101 (50.2) | | 12078 (50.3) | | 11010 (50.3) | | 9663 (50.2) | | 0.444 |
|  | Laparoscopic | 12475 (49.1) | 10995 (49.8) | | 11923 (49.7) | | 10896 (49.7) | | 9594 (49.8) | |  |
| Operation time, min (median [IQR]) |  | 197.00 [151.00, 254.00] | 198.00 [151.00, 255.00] | | 199.00 [152.00, 256.00] | | 197.00 [152.00, 254.00] | | 199.00 [152.00, 254.00] | | 0.062 |
| Estimated blood loss, mL (median [IQR]) |  | 67.00 [20.00, 187.50] | 62.00 [20.00, 180.00] | | 65.00 [20.00, 183.00] | | 60.00 [20.00, 178.00] | | 60.00 [20.00, 180.00] | | <0.001 |
| 30-Day mortality | No | 25269 (99.4) | 21949 (99.3) | | 23879 (99.5) | | 21786 (99.5) | | 19162 (99.5) | | 0.077 |
|  | Yes | 156 (0.6) | 147 (0.7) | | 121 (0.5) | | 120 (0.5) | | 95 (0.5) | |  |
| Surgical mortality | No | 25144 (98.9) | 21838 (98.8) | | 23766 (99.0) | | 21671 (98.9) | | 19068 (99.0) | | 0.233 |
|  | Yes | 281 (1.1) | 258 (1.2) | | 235 (1.0) | | 235 (1.1) | | 189 (1.0) | |  |
| Morbidity with CD classification of III or more | No | 24207 (95.2) | 21022 (95.2) | | 22888 (95.4) | | 20950 (95.6) | | 18365 (95.4) | | 0.121 |
|  | Yes | 1216 (4.8) | 1070 (4.8) | | 1107 (4.6) | | 954 (4.4) | | 888 (4.6) | |  |
| Anastomotic leakage | No | 25018 (98.4) | 21754 (98.5) | | 23617 (98.4) | | 21555 (98.4) | | 18952 (98.4) | | 0.992 |
|  | Yes | 402 (1.6) | 339 (1.5) | | 380 (1.6) | | 347 (1.6) | | 301 (1.6) | |  |
|  |  |  | |  | |  | |  | |  |  |

BMI, body mass index; COPD, chronic obstructive pulmonary disease; ASA_PS, American Society of Anesthesiologists Performance Status; PT-INR, prothrombin time-international normalized ratio; APTT, activated partial thromboplastin time; BUN, blood urea nitrogen; IQR, interquartile range; CD, Clavien-Dindo

| **Table 3. Risk-Adjusted Model for Surgical Mortality** | | | | | | | |
| --- | --- | --- | --- | --- | --- | --- | --- |
|  |  | **Odds ratio** | **2.5%** | | **97.5%** | | ***P*** |
| Day of the week | Monday | Reference |  | |  | |  |
|  | Tuesday | 1.010 | 0.841 | | 1.213 | | 0.915 |
|  | Wednesday | 0.871 | 0.724 | | 1.048 | | 0.144 |
|  | Thursday | 0.924 | 0.767 | | 1.114 | | 0.408 |
|  | Friday | 0.873 | 0.716 | | 1.063 | | 0.176 |
| Year | 2012 | Reference |  | |  | |  |
|  | 2013 | 1.171 | 0.946 | | 1.450 | | 0.147 |
|  | 2014 | 1.331 | 1.079 | | 1.642 | | 0.008 |
|  | 2015 | 1.167 | 0.942 | | 1.447 | | 0.157 |
|  | 2016 | 1.047 | 0.839 | | 1.307 | | 0.685 |
|  | 2017 | 1.179 | 0.948 | | 1.467 | | 0.139 |
| Age | 18–59 | Reference |  | |  | |  |
|  | 60–64 | 1.057 | 0.712 | | 1.568 | | 0.784 |
|  | 65–69 | 1.141 | 0.807 | | 1.612 | | 0.456 |
|  | 70–74 | 1.261 | 0.905 | | 1.756 | | 0.170 |
|  | 75–79 | 1.764 | 1.287 | | 2.417 | | 0.000 |
|  | 80– | 2.629 | 1.947 | | 3.550 | | 0.000 |
| Sex | Female | Reference |  | |  | |  |
|  | Male | 1.522 | 1.326 | | 1.746 | | 0.000 |
| BMI, kg/m^2^ | normal (18.5 ≤ BM I < 25) | Reference |  | |  | |  |
|  | underweight (BMI < 18.5) | 1.212 | 1.046 | | 1.404 | | 0.010 |
|  | overweight (BMI ≥ 25) | 1.029 | 0.863 | | 1.227 | | 0.750 |
| Activities of daily living | independent | Reference |  | |  | |  |
|  | dependent | 2.974 | 2.577 | | 3.432 | | 0.000 |
| Brinkman index | 0 | Reference |  | |  | |  |
|  | <400 | 0.981 | 0.773 | | 1.244 | | 0.874 |
|  | ≥400 | 0.885 | 0.743 | | 1.053 | | 0.168 |
| COPD | No | Reference |  | |  | |  |
|  | yes | 1.313 | 1.008 | | 1.710 | | 0.044 |
| Dyspnea | No | Reference |  | |  | |  |
|  | Yes | 1.890 | 1.492 | | 2.394 | | 0.000 |
| Angina | No | Reference |  | |  | |  |
|  | Yes | 1.037 | 0.702 | | 1.531 | | 0.856 |
| Loss of body weight | No | Reference |  | |  | |  |
|  | Yes | 1.742 | 1.447 | | 2.096 | | 0.000 |
| Coagulation disorder | No | Reference |  | |  | |  |
|  | Yes | 1.214 | 0.972 | | 1.515 | | 0.087 |
| ASA_PS | ASA_PS_1-2 | Reference |  | |  | |  |
|  | ASA_PS_3-5 | 2.133 | 1.860 | | 2.447 | | 0.000 |
| T category | T0, Tis, 1, 2 | Reference |  | |  | |  |
|  | T3, 4a, 4b | 1.299 | 1.048 | | 1.610 | | 0.017 |
|  | TX | 1.279 | 0.497 | | 3.288 | | 0.610 |
| N category | N0 | Reference |  | |  | |  |
|  | N1a, 1b, 1c, 2a, 2b | 1.216 | 1.058 | | 1.398 | | 0.006 |
|  | NX | 2.929 | 2.136 | | 4.016 | | 0.000 |
| M category | M0 | Reference |  | |  | |  |
|  | M1a, M1b | 3.604 | 3.132 | | 4.145 | | 0.000 |
| Surgical approach | Open | Reference |  | |  | |  |
|  | Laparoscopic | 0.533 | 0.458 | | 0.620 | | 0.000 |
| White blood cell count, /μL | Normal (3500–9000) | Reference |  | |  | |  |
|  | Low (<3500) | 1.450 | 1.089 | | 1.930 | | 0.011 |
|  | High (>9000) | 2.580 | 2.223 | | 2.995 | | 0.000 |
| Platelet count, ×10^4^/μL | Normal (15–35） | Reference |  | |  | |  |
|  | Low (<15) | 2.035 | 1.651 | | 2.509 | | 0.000 |
|  | High (>35） | 0.755 | 0.631 | | 0.902 | | 0.002 |
| PT-INR | Normal (0.9–1.1） | Reference |  | |  | |  |
|  | Low (<0.9) | 0.626 | 0.286 | | 1.374 | | 0.243 |
|  | High (>1.1) | 1.746 | 1.503 | | 2.029 | | 0.000 |
| APTT, sec | Normal (30–40) | Reference |  | |  | |  |
|  | Low (<30) | 0.888 | 0.753 | | 1.047 | | 0.157 |
|  | High (>40) | 1.439 | 1.160 | | 1.785 | | 0.001 |
| BUN, mg/dL | Normal (8–20) | Reference |  | |  | |  |
|  | Low (<8) | 1.400 | 1.093 | | 1.792 | | 0.008 |
|  | High (>20) | 1.191 | 1.013 | | 1.399 | | 0.034 |
| Creatinine, mg/dL | Normal (≤2) | Reference |  | |  | |  |
|  | High (>2) | 2.182 | 1.625 | | 2.931 | | 0.000 |
|  |  |  | |  | |  |  |

BMI, body mass index; COPD, chronic obstructive pulmonary disease; ASA_PS, American Society of Anesthesiologists Performance Status; PT-INR, prothrombin time-international normalized ratio; APTT, activated partial thromboplastin time; BUN, blood urea nitrogen
